# Coping strategies of health professionals suspected or confirmed to have COVID-19 in a teaching maternity hospital

**DOI:** 10.47626/1679-4435-2025-1386

**Published:** 2025-07-13

**Authors:** Romanniny Hévillyn Silva Costa Almino, Juliana Dantas de Araújo Santos Camargo, Paula Laís Padilha Martinho, Cybelle Dutra da Silva, Rebecca Stefany da Costa Santos, Janice França de Queiroz, Sávio Ferreira Camargo, Richardson Augusto Rosendo da Silva

**Affiliations:** 1 Maternidade Escola Januário Cicco (MEJC), Empresa Brasileira de Serviços Hospitalares (Ebserh), Universidade Federal do Rio Grande do Norte (UFRN), Natal, RN, Brazil; 2 Instituto Federal do Rio Grande do Norte, Natal, RN, Brazil; 3 Programa de Pós-Graduação em Ciências da Saúde, UFRN, Natal, RN, Brazil; 4 Departamento de Enfermagem, UFRN, Natal, RN, Brazil

**Keywords:** psychological adaptation, occupational stress, health personnel, COVID-19, adaptação psicológica, estresse ocupacional, pessoal de saúde, covid-19

## Abstract

**Introduction:**

The COVID-19 pandemic impacted the physical and mental health of health professionals.

**Objectives:**

To assess the coping strategies employed by health professionals in response to the suspicion or confirmation of infection with the novel coronavirus (severe acute respiratory syndrome coronavirus 2) according to their sociodemographic, clinical-epidemiological, and occupational profiles.

**Methods:**

This cross-sectional study was conducted from March 2020 to January 2021 at a teaching maternity hospital located in the Northeast region of Brazil and included 188 health professionals. The Coping Strategies Inventory was used to assess confrontation, problem-solving, distancing, self-care, acceptance of responsibility, positive reappraisal, escape-avoidance, and social support. Cronbach’s alpha, Mann-Whitney, Kruskal-Wallis, and post-hoc tests were applied (α = 5%).

**Results:**

Social support (1.28 ± 0.70) and problem-solving (1.25 ± 0.68) were the most frequently adopted coping strategies. Women scored significantly higher than men in social support (1.33 vs. 0.83; p = 0.046), escape-avoidance (1.00 vs. 0.50; p = 0.036), and positive reappraisal (1.22 vs. 0.95; p = 0.009). Professionals who reported adopting preventive measures in the workplace had higher scores in social support (p = 0.016) and positive reappraisal (p = 0.016).

**Conclusions:**

Social support and problem-solving were the main coping strategies used in the face of suspected or confirmed COVID-19. Understanding the needs and experiences of health professionals during crises is essential for developing targeted interventions that respect the specific characteristics of each group.

## Abstract (pt)

**Introdução:**

A pandemia de covid-19 impactou a saúde física e mental dos profissionais de saúde.

**Objetivos:**

Avaliar as estratégias de enfrentamento da suspeição/confirmação do novo coronavírus (síndrome respiratória aguda grave do coronavírus 2) de profissionais de saúde, nos perfis sociodemográfico, clínico-epidemiológico e ocupacional.

**Métodos:**

Este estudo transversal foi conduzido de março de 2020 a janeiro de 2021 em uma maternidade-escola localizada no Nordeste do Brasil e incluiu 188 profissionais de saúde. Foi utilizado o Inventário de Estratégias de Enfrentamento para avaliar: confronto, resolução de problemas, afastamento, autocuidado, aceitação de responsabilidades, reavaliação positiva, fuga-esquiva e suporte social. Foram aplicados os testes de Cronbach, Mann-Whitney, Kruskal-Wallis e comparações *post-hoc* (α = 5%).

**Resultados:**

Suporte social (1,28 ± 0,70) e resolução de problemas (1,25 ± 0,68) foram as estratégias mais utilizadas. Mulheres apresentaram escores significativamente maiores do que homens em suporte social (1,33 vs. 0,83; p = 0,046), fuga-esquiva (1,00 vs. 0,50; p = 0,036) e reavaliação positiva (1,22 vs. 0,95; p = 0,009). Profissionais que relataram a adoção de medidas preventivas no ambiente de trabalho obtiveram escores mais elevados em suporte social (p = 0,016) e reavaliação positiva (p = 0,016).

**Conclusões:**

Suporte social e resolução de problemas foram as principais estratégias de enfrentamento adotadas diante da suspeita ou diagnóstico de covid-19. A compreensão das necessidades e experiências dos profissionais de saúde em contextos de crise é essencial para o aprimoramento de intervenções, respeitando as especificidades de cada grupo.

## INTRODUCTION

The COVID-19 pandemic, caused by the novel coronavirus (severe acute respiratory syndrome coronavirus 2 [SARS-CoV-2]), was first reported on December 12, 2019, in the city of Wuhan, Hubei Province, China. Due to its rapid spread, the World Health Organization (WHO) declared it a Public Health Emergency of International Concern on January 30, 2020.^1^

Because the virus is highly infectious, health care systems had to be reorganized to accommodate the growing number of critically ill patients. These efforts included strengthening the surveillance system and intensifying prevention and control actions to curb the spread of the pandemic.^2^

Amid this climate of uncertainty, health professionals were faced with the challenge of caring for critically ill patients, managing overcrowded facilities, and coping with high mortality rates.^3^ The stress and pressure inherent to their professional duties, combined with the constant risk of infection, created conditions that significantly increased the likelihood of developing serious mental health problems.^4^

The rapid clinical deterioration of patients with COVID-19 increased the risk of infection among health professionals in their work environments. This occupational group was considered one of the most vulnerable to contracting the virus, which contributed to growing skepticism about the effectiveness of preventive measures. These perceptions were largely driven by shortages of personal protective equipment, prolonged exposure to large numbers of infected patients, and inadequate training in infection prevention and control protocols for COVID-19.^5^

Physical distancing and social isolation of patients suspected or confirmed to have COVID-19 aimed primarily to contain the spread of the virus. However, the removal of health professionals from their work duties — marked by an abrupt transition from a structured routine to mandatory isolation — led to significant changes that may have contributed to the onset of stress and anxiety symptoms. These effects were influenced both by the new home environment and by the psychological impact of the disease itself.^4^

Similar challenges experienced during the COVID-19 outbreak led to various psychological issues and emotional disorders among health professionals working on the front lines of the pandemic response. Evidence suggests that changes imposed on work processes increased the burden of responsibility in task execution and had psychological repercussions within the family environment.^6^

During the pandemic, a high prevalence of posttraumatic stress, anxiety, and depression symptoms was observed among professionals working in emergency services. However, not all individuals exposed to intense negative impacts or crisis situations develop these symptoms, with resilience being recognized as an important protective factor.^7^

Therefore, assessing the coping strategies adopted by health professionals in response to suspected or confirmed SARS-CoV-2 infection — considering their sociodemographic, clinical-epidemiological, and occupational profiles — can inform the development of targeted support and occupational health interventions during crisis situations such as the COVID-19 pandemic.

## METHODS

This was a cross-sectional study conducted at a public teaching maternity hospital in Northeastern Brazil, which serves as a referral center for high-risk pregnancies, women’s health, and gynecological surgery. At the time of the study, the hospital employed 785 health professionals. The sample included all professionals who were reported as suspected or confirmed COVID-19 cases between March 2020 and January 2021 (n = 188). Professionals who required hospitalization due to severe illness or who progressed to death were excluded from the study.

The research project was submitted to the Research Ethics Committee of the Onofre Lopes University Hospital, Universidade Federal do Rio Grande do Norte, and was approved under CAAE No. 33482620.4.0000.5292. The study was conducted in accordance with the Declaration of Helsinki and Resolution No. 466/2012 of the Brazilian National Health Council.

Participants were identified by the Occupational Health and Workplace Safety Service and invited to take part in the study. The invitation, along with the Informed Consent Form and the research instruments — including a sociodemographic, clinical-epidemiological, and occupational questionnaire as well as the Coping Strategies Inventory (CSI) — was sent via email. Data collection was conducted through the Google Forms platform.

To assess coping strategies related to COVID-19, participants completed the CSI, a tool designed to identify strategies adopted in stressful situations, based on actions taken during the period of work leave due to suspected and/or confirmed infection. The inventory consists of 66 items distributed across eight coping factors, categorized as follows: problem-focused coping (confrontation and problem-solving), emotion-focused coping (distancing, self-care, acceptance of responsibility, positive reappraisal, and escape-avoidance), and coping strategies focused on both problem and emotion (social support).^8^

Sociodemographic variables included gender, age, and marital status. Clinical-epidemiological data encompassed COVID-19 diagnosis confirmation, duration of medical leave, and history of household or occupational contact with infected individuals. The occupational profile was assessed based on professional category, use of personal protective equipment, and implementation of preventive measures in the workplace.

For statistical analysis, the Shapiro-Wilk test was applied to assess the normality of continuous variables. Descriptive statistics included measures of central tendency (mean and median) and dispersion (standard deviation and 25th and 75th percentiles). Mean scores were calculated for each of the eight factors of CSI. Factor scores ranged from 0 (“did not use”) to 3 (“used frequently”).

Mann-Whitney and Kruskal-Wallis tests were used to compare continuous variable distributions between two or more groups. When comparisons involved more than two groups, post-hoc analyses were performed using Dunn’s test^9^ with Bonferroni correction for multiple comparisons. Cronbach’s alpha coefficient was calculated to assess the internal consistency (reliability) of the instrument. A significance level of α = 5% was adopted for all analyses. Statistical analyses were conducted using IBM SPSS Statistics for Windows, version 28 (IBM Corp., Armonk, NY, USA).

## RESULTS

The study included 188 participants. Most of the participants were women, between 30 and 39 years old, and reported being married or in a stable union (Table 1).

**Table 1:** Sociodemographic profile of health professionals (n = 188), Natal, Rio Grande do Norte, Brazil, 2021

Variables	n (%)
Gender, n (%)	
Female	164 (87.2)
Male	24 (12.8)
Age group, years (n [%])	
20-29	18 (9.6)
30-39	90 (47.9)
40-49	52 (27.7)
50 or more	28 (14.9)
Marital status, n (%)	
Married or in a stable union	131 (69.7)
Single, separated, or divorced	57 (30.3)

Categorical data are expressed as absolute frequency (n) and relative frequency (%).

The scale demonstrated good overall internal consistency (α = 0.872). The internal consistency of the individual factors ranged from 0.50 (self-control) to 0.85 (positive reappraisal). Among the coping factors assessed, social support had the highest mean score, while confrontation had the lowest (Table 2).

**Table 2:** Coping Strategy Inventory factor scores (n = 188), Natal, Rio Grande do Norte, Brazil, 2021

Factors	Mean	Standard deviation	Median	Percentiles (25th–75th)	α
Confrontation (6 items)	0.40	0.35	0.33	(0.17-0.67)	0.54
Distancing (7 items)	0.69	0.40	0.71	(0.43-1.00)	0.60
Self-control (5 items)	0.93	0.46	1.00	(0.60-1.20)	0.50
Social support (6 items)	1.28	0.70	1.33	(0.67-1.83)	0.76
Acceptance of responsibility (7 items)	0.69	0.48	0.71	(0.29-1.00)	0.70
Escape-avoidance (2 items)	1.23	0.90	1.00	(0.50-2.00)	0.73
Problem-solving (4 items)	1.25	0.68	1.25	(0.75-1.75)	0.74
Positive reappraisal (9 items)	1.20	0.64	1.22	(0.67-1.67)	0.85

Regarding the sociodemographic profile, female participants had significantly higher scores than male participants for the coping strategies of social support (1.33 vs. 0.83; p = 0.046), escape-avoidance (1.00 vs. 0.50; p = 0.036), and positive reappraisal (1.22 vs. 0.95; p = 0.009). With respect to age group, statistically significant differences were observed in four factors: confrontation, acceptance of responsibility, escape-avoidance, and positive reappraisal. In all cases, participants aged 50 years or older had lower median scores compared to the younger age groups (Table 3).

**Table 3:** Sociodemographic profile and coping strategy factors among health professionals with suspected and/or confirmed COVID-19 (n = 188), Natal, Rio Grande do Norte, Brazil, 2021

	Confrontation	Distancing	Self-control	Social support	Acceptance of responsibility	Escape-avoidance	Problem-solving	Positive reappraisal
Gender, n (%)								
Female	0.33	0.57	1.00	1.33	0.71	1.00	1.25	1.22
Male	0.42	1.00	0.90	0.83	0.50	0.50	1.00	0.95
p-value*	0.834	0.143	0.263	**0.046**	0.187	**0.036**	0.059	**0.009**
Age group, years [n (%)]								
20-29	0.37	0.71	1.00	1.33	0.86	1.25	1.38	1.00
30-39	0.33	0.57	1.00	1.33	0.71	1.50	1.25	1.22
40-49	0.33	0.86	1.00	1.33	0.71	1.00	1.50	1.33
50 or more	0.17	0.57	0.60	1.00	0.43	0.50	1.00	0.89
p-value^†^	**0.014**	0.065	0.082	0.301	**0.007**	**0.013**	0.054	**0.041**
Marital status, n (%)								
Married/stable union	0.33	0.71	1.00	1.17	0.71	1.00	1.25	1.22
Single, separated, or divorced	0.33	0.57	1.00	1.50	0.71	1.00	1.25	1.11
p-value*	0.508	0.169	0.524	0.212	0.894	0.696	0.640	0.781

Continuous variables are expressed as medians. Bold values indicate statistical significance at the 5% level.

* p-value obtained using the Mann-Whitney *U* test;

† p-value obtained using the Kruskal-Wallis test.

In the post-hoc comparisons, statistically significant differences were confirmed for the following factors and age groups: confrontation – 50 years or more vs. 40-49 years (p = 0.007); acceptance of responsibility – 50 years or more vs. 40-49 years (p = 0.009) and 50 years or more vs. 20-29 years (p = 0.033); escape-avoidance – 50 years or more vs. 30-39 years (p = 0.007); and positive reappraisal – 50 years or more vs. 40-49 years (p = 0.028) (Table 3).

Participants with confirmed COVID-19 diagnoses showed higher scores in the factors of self-control, social support, acceptance of responsibility, escape-avoidance, and positive reappraisal compared to those with suspected but unconfirmed cases. Among professionals on leave from work, those with absences longer than 14 days had higher scores in the social support factor. In addition, those who were absent for more than 10 days scored higher in the positive reappraisal factor (Table 4).

**Table 4:** Clinical-epidemiological profile and coping strategy factors among health professionals with suspected and/or confirmed COVID-19 (n = 188), Natal, Rio Grande do Norte, Brazil, 2021

Variables	Confrontation	Distancing	Self-control	Social support	Acceptance of responsibility	Escape-avoidance	Problem-solving	Positive reappraisal
COVID-19 diagnosis, n (%)								
Yes	0.33	0.71	1.00	1.67	0.86	1.50	1.25	1.33
No	0.33	0.57	0.80	1.17	0.70	1.00	1.25	1.00
p-value*	0.246	0.241	**0.036**	**< 0.01**	**0.026**	**0.023**	0.115	**0.001**
Length of leave, years [n (%)]								
Up to 7	0.33	0.71	1.00	1.00	0.57	1.00	1.00	1.00
8 to 10	0.33	0.57	0.80	1.17	0.71	1.50	1.25	1.00
11 to 14	0.33	0.71	1.00	1.33	0.71	1.00	1.25	1.33
Over 14	0.33	0.57	1.00	1.83	0.86	1.50	1.25	1.56
p-value^†^	0.417	0.989	0.511	**0.001**	0.166	0.078	**0.011**	**0.001**
Household contact with suspected COVID-19 case, n (%)								
Yes	0.33	0.57	1.00	1.33	0.71	1.50	1.25	1.33
No	0.33	0.71	1.00	1.17	0.57	1.00	1.00	1.00
p-value*	**0.035**	0.185	0.472	0.105	0.063	**0.007**	0.213	**0.002**
Workplace contact with confirmed COVID-19 case, n (%)								
Yes	0.50	0.71	1.00	1.33	0.71	1.00	1.25	1.33
No	0.33	0.57	0.80	1.17	0.57	1.00	1.00	1.00
p-value*	**0.002**	0.307	**0.002**	0.103	**0.035**	0.182	**0.049**	**0.019**

Continuous variables are expressed as medians. Bold values indicate statistical significance at the 5% level.

* p-value obtained using the Mann-Whitney *U* test;

† p-value obtained using the Kruskal-Wallis test.

In the post-hoc comparisons, these differences were confirmed for the following factors and durations of work leave: social support – over 14 days vs. 8 to 10 days (p = 0.027), over 14 days vs. 11 to 14 days (p = 0.025), and over 14 days vs. up to 7 days (p = 0.001); problem-solving – up to 7 days vs. 11 to 14 days (p = 0.014); and positive reappraisal – up to 7 days vs. over 14 days (p = 0.001) (Table 4).

Professionals who reported household contact with individuals suspected of having COVID-19 showed higher scores in the escape-avoidance and positive reappraisal coping strategies. Those who reported workplace contact with colleagues diagnosed with COVID-19 had higher scores in the confrontation, self-control, acceptance of responsibility, problem-solving, and positive reappraisal strategies (Table 4).

Within the occupational profile, statistically significant differences between professional categories were observed only in the distancing factor, with nursing technicians or assistants showing lower median scores compared to other categories. Post-hoc testing confirmed this difference specifically between nursing technicians/aides and registered nurses. Additionally, professionals who reported not using preventive measures in the workplace had higher scores in the escape-avoidance factor. In contrast, those who reported the presence of preventive measures at their workplace showed higher scores in the social support and positive reappraisal strategies (Table 5).

**Table 5:** Occupational profile and coping strategy factors among health professionals with suspected and/or confirmed COVID-19 (n = 188), Natal, Rio Grande do Norte, Brazil, 2021

Variables	Confrontation	Distancing	Self-control	Social support	Acceptance of responsibility	Escape-avoidance	Problem-solving	Positive reappraisal
Professional category, n (%)								
Nursing technician or aide	0.33	0.57	0.80	1.17	0.71	1.00	1.13	1.22
Registered nurse	0.33	0.71	1.00	1.33	0.71	1.00	1.25	1.17
Physician	0.50	0.71	1.00	1.33	0.79	1.00	1.50	1.28
Other professional category	0.33	0.71	1.00	1.59	0.57	1.00	1.13	1.00
p-value*	0.229	**0.018**	0.087	0.696	0.096	0.245	0.053	0.235
Use of preventive measures by professional, n (%)								
Yes	0.33	0.71	1.00	1.33	0.71	1.00	1.25	1.22
No	0.83	0.57	0.60	1.83	0.71	2.50	1.00	1.33
p-value^†^	0.351	0.355	0.139	0.764	0.872	**0.046**	0.294	0.776
Presence of preventive measures in the workplace, n (%)								
Yes	0.33	0.71	1.00	1.33	0.71	1.00	1.25	1.28
No	0.33	0.57	0.80	1.17	0.71	1.00	1.00	1.00
p-value*	0.655	0.238	0.451	**0.016**	0.509	0.717	0.146	**0.016**

Continuous variables are expressed as medians. Bold values indicate statistical significance at the 5% level.

* p-value obtained using the Kruskal-Wallis test;

† p-value obtained using the Mann-Whitney *U* test.

## DISCUSSION

The COVID-19 pandemic had a significant impact on the physical and mental health of health professionals. Those working on the front lines were more vulnerable to illness, both due to direct exposure to the infectious agent and to the negative mental health effects associated with high levels of responsibility and workload.^10^ This study underscores the importance of understanding the coping strategies adopted by professionals with suspected or confirmed infection considering their sociodemographic, clinical-epidemiological, and occupational profiles. Such understanding is essential to support the development and enhancement of targeted occupational health interventions.

The main finding of this study indicates that social support and problem-solving were the most frequently used coping strategies among health professionals with suspected or confirmed SARS-CoV-2 infection. A similar result was reported in a study conducted in Iran, where problem-focused coping was the most commonly adopted strategy, used by approximately 80% of participants to manage stress.^11^ In Germany, 3 strategies were frequently mentioned: social support, hobbies/leisure activities, and mental strategies.^12^

These findings underscore the importance of promoting the adoption of these strategies among professionals required to take medical leave. Since mental health programs and individual coping strategies tend to be more effective when integrated into routines in a continuous and structured manner, these practices should be encouraged not only during times of crisis but also in non-pandemic periods.^13^

In contexts of increased workload, social support from family and friends can serve as a protective factor against psychological stress. Managers can implement organizational measures that facilitate such support, such as providing videocommunication equipment during breaks, enabling professionals to stay in touch with their families and alleviate their concerns.^14^

Regarding the sociodemographic profile, social support, escape-avoidance, and positive reappraisal strategies were more frequently used by female participants. Similar findings were reported in another study which also identified higher coping strategy scores among women.^15^

In Brazil, women represent the majority of the health care workforce. However, this pattern is not directly attributable to the COVID-19 pandemic since the feminization of health care occupations was already well established prior to the health crisis. This predominance is closely linked to a sociocultural construct that historically associates caregiving and service-oriented roles with maternal vocation and the extension of domestic labor.^16^ The predominance of women in the health care sector influences the coping strategies adopted, given the cumulative workload, increased patient contact, and heightened risk of exposure to infection. These factors are intensified by the nature of occupations predominantly held by women.^17^

In addition, the advanced age of participants appeared to influence coping strategy use since individuals aged 50 and older showed lower scores across all factors analyzed. Similar results were reported in studies conducted in Yemen and Pakistan, which also found lower use of coping strategies among older participants.^15^,^18^ Our findings are consistent with other research suggesting that health professionals with less experience tend to exhibit greater coping abilities.^18^,^19^ This may be explained by a limited awareness of potential consequences.

Participants with a confirmed COVID-19 diagnosis showed higher scores in the coping strategies of self-control, social support, acceptance of responsibility, escape-avoidance, and positive reappraisal compared to those with only suspected cases. These findings suggest that, upon receiving a diagnosis, individuals adopted behaviors that were positive both individually and collectively, promoting adherence to isolation measures and self-care during the recovery period.^20^

As a reflection of this acceptance process, professionals diagnosed with COVID-19 demonstrated behaviors similar to those reported in a national study, which found that a large portion of the population adhered to restriction and isolation measures across all regions of the country — with higher adherence in the Southeast and lower in the Midwestern and Northern regions.^21^

Identifying which occupations carry a higher risk of exposure to COVID-19 is essential. Then, risk management strategies and training programs can be developed and tailored to workers. Furthermore, response plans must be prioritized since an infected professional could potentially transmit the virus to colleagues and family members.^22^

A study conducted in Scotland found that a significant proportion of COVID-19-related hospital admissions consisted of family members of health professionals. Although they represent a relatively small share of the working-age population, health professionals and their households had a substantial impact on hospitalization rates.^23^

Another noteworthy finding concerns the duration of work leave. Social support, problem-solving, and positive reappraisal strategies were more frequently used by professionals with longer periods of leave. In summary, this result may reflect a positive perception of time off from work, seen as an effective coping mechanism in the context of COVID-19.

Leave and isolation measures have been widely recognized as effective strategies for containing the spread of the disease, both in the community and among health professionals.^21^-^23^ This reflects a general consensus among these professionals regarding the importance and effectiveness of such practices.

However, recognition of their effectiveness is not always sufficient, as the removal of professionals from the workplace has a dual impact on coping strategies: it affects the individual who is absent, and it also places additional strain on colleagues, who face an increased workload. In this context, ensuring appropriate working conditions is essential for preserving the physical and mental health of health professionals during the pandemic.^24^

Higher scores in escape-avoidance and positive reappraisal strategies were also observed among professionals who reported household contact with individuals suspected or confirmed to have COVID-19. In contrast, those with workplace exposure more frequently adopted confrontation, self-control, acceptance of responsibility, problem-solving, and positive reappraisal strategies. These findings suggest that, in the professional setting, there is a tendency to adopt more active coping strategies, reflecting stricter control measures and response protocols compared to those employed in the household context.

The nature of health care work not only increases the risk of exposure to infectious diseases but also positions health professionals as potential links in the chain of community and hospital transmission—especially when they continue working even after receiving a positive diagnosis. Understanding the transmission cycle of infectious diseases and identifying the most vulnerable professional categories have been essential for enabling rapid responses to COVID-19 by health authorities, while also contributing to risk mitigation and the prevention of future outbreaks.^21^-^23^ An illustrative example can be found during the 2009 H1N1 pandemic, when a study estimated that approximately 8 million health professionals in the United States were infected while performing their duties, potentially transmitting the virus to about 7 million coworkers and numerous household contacts.^25^ Therefore, understanding the impact of occupational exposure — including the number of workers potentially affected and the specific roles involved — is crucial for developing effective prevention strategies.^22^

Regarding professional category, nursing technicians and nursing aides showed greater use of the distancing strategy when faced with suspected or confirmed COVID-19. This suggests a coping approach grounded in acceptance of isolation, without denial or minimization of the situation. Nursing professionals are well-known to be among the most vulnerable to illness caused by the novel coronavirus due to their role in providing direct and continuous patient care. Furthermore, nurses are the largest professional group in health care, particularly in Brazil.^26^ Data from the Brazilian Federal Nursing Council (Conselho Federal de Enfermagem, COFEN), published on July 11, 2021, reported that 57,580 nursing professionals had been infected with the novel coronavirus up to that date.^27^

Studies suggest that nursing professionals have prior experience in managing infectious diseases and in assuming risks — including those related to their own health — due to the nature of their work and the social commitment it entails.^26^ These factors may have contributed to a more effective coping response among this group. Additionally, other studies have shown that health professionals expressed concern about the possibility of transmitting the disease to family members and close contacts, which may have encouraged a more responsible attitude and greater adherence to precautionary and isolation measures, reinforcing a positive coping approach in response to the situation.^6^

Various measures are recommended for health professionals to prevent COVID-19, including frequent hand hygiene, proper use of personal protective equipment, monitoring for symptoms suggestive of the disease, and adherence to social distancing. Studies have shown that most health professionals complied with individual preventive measures, reinforcing both self-care and collective protection.^28^

In this study, although most participants reported believing they had adhered to precautionary measures, health professionals on leave due to suspected or confirmed COVID-19 who reported not using individual preventive measures showed greater use of the escape-avoidance coping strategy. This may suggest that these individuals either failed to adopt preventive strategies to avoid exposure or faced gaps in the implementation of protective practices. A study conducted in Thailand demonstrated that, although participants had satisfactory knowledge of COVID-19 transmission routes and symptoms, deficiencies were identified in both knowledge and practice — particularly regarding the correct steps for hand hygiene and the minimum recommended distance for social distancing.^28^

With regard to workplace preventive measures, participants who reported the presence of such actions demonstrated greater use of social support and positive reappraisal coping strategies. Studies emphasize the importance of implementing coping strategies for the COVID-19 pandemic within the work environment — not only to prevent disease transmission, but also to promote the mental health of health professionals. For these actions to be effective, the integrated participation of multiple institutional stakeholders is essential, including administrators, patients, occupational health teams, mental health teams, engineering departments, hospital infection control committees, and educational departments.^28^-^30^

In addition, the support provided by occupational health teams to professionals — even during periods of leave due to suspected or confirmed COVID-19 — is essential. This follow-up enables appropriate clinical monitoring and offers guidance on home isolation protocols and the appropriate timing for returning to work activities. The availability of diagnostic testing also plays a crucial role as it facilitates faster diagnosis, contributing both to proper clinical management and to the prevention of coronavirus transmission in the workplace.^29^

Occupational health and mental health teams within the institution also play a key role in promoting the mental well-being of health professionals and in preventing illness during the pandemic, including during periods of isolation. The range of emotions triggered by uncertainty regarding the diagnosis and prognosis of the disease can lead to significant psychological distress, making support and follow-up by in-house professionals essential for fostering a more positive coping process.^30^

This study has a limitation related to its cross-sectional design, which does not allow for the establishment of causal relationships between leave due to suspected or confirmed COVID-19 and the coping strategies adopted. Nevertheless, the research makes an important contribution by examining these strategies in light of the sociodemographic, clinical-epidemiological, and occupational profiles of health professionals within an emergency crisis context, offering valuable insights for improving occupational health interventions. The relevance of the findings could be enhanced by conducting longitudinal follow-up with the sample, enabling a comparative analysis of impacts in the pre- and post-pandemic periods.

## CONCLUSIONS

The most frequently used coping strategies among health professionals were based on social support and problem-solving. The findings of this study highlight the importance of understanding the needs and experiences of these workers in crisis contexts, in order to support and improve occupational health interventions that are tailored to the specific characteristics of each professional group.

Such interventions should prioritize the well-being of health professionals by addressing health promotion, disease prevention, and treatment. Both local and national support are essential to ensuring comprehensive health care that promotes quality of life in the workplace and protects mental health.
